# Preventive Treatment for Household Contacts of Drug-Susceptible Tuberculosis Patients

**DOI:** 10.3390/pathogens11111258

**Published:** 2022-10-29

**Authors:** Nicole Salazar-Austin, Christiaan Mulder, Graeme Hoddinott, Theresa Ryckman, Colleen F. Hanrahan, Kavindhran Velen, Lucy Chimoyi, Salome Charalambous, Violet N Chihota

**Affiliations:** 1 Department of Pediatrics, Johns Hopkins University, Baltimore, MD 21287, USA; 2 Department of TB Elimination and Health System Innovations, KNCV Tuberculosis Foundation, 2516 The Hague, The Netherlands; 3 Amsterdam Institute for Global Health and Development, Amsterdam University Medical Centres, 1105 Amsterdam, The Netherlands; 4 Desmond Tutu TB Centre, Department of Paediatrics and Child Health, Faculty of Medicine and Health Sciences, Stellenbosch University, Cape Town 7500, South Africa; 5 Department of Epidemiology, Johns Hopkins Bloomberg School of Public Health, Baltimore, MD 21287, USA; 6 Faculty of Medicine and Health, The Central Clinical School, The University of Sydney, 90-92 Parramatta Road, Sydney, NSW 2006, Australia; 7 The Aurum Institute, Parktown, Johannesburg 2000, South Africa; 8 School of Public Health, University of Witwatersrand, Johannesburg 2000, South Africa; 9 Division of Epidemiology of Microbial Diseases, Yale School of Public Health, New Haven, CT 06510, USA; 10 Department of Medicine, Division of Infectious Diseases, School of Medicine, Vanderbilt University, Nashville, TN 37240, USA

**Keywords:** TB infection, household contacts, TB preventive treatment

## Abstract

People who live in the household of someone with infectious pulmonary tuberculosis are at a high risk of tuberculosis infection and subsequent progression to tuberculosis disease. These individuals are prioritized for contact investigation and tuberculosis preventive treatment (TPT). The treatment of TB infection is critical to prevent the progression of infection to disease and is prioritized in household contacts. Despite the availability of TPT, uptake in household contacts is poor. Multiple barriers prevent the optimal implementation of these policies. This manuscript lays out potential next steps for closing the policy-to-implementation gap in household contacts of all ages.

## 1. Introduction

Tuberculosis (TB) is a leading infectious cause of mortality globally that caused an estimated 1.5 million deaths in 2020 [1]. TB preventive treatment (TPT) is an effective and cost-effective intervention to prevent progression from infection to disease and to reduce mortality. The World Health Organization (WHO) has recently expanded the guidelines to provide TPT to household contacts of all ages [2]. To add momentum and urgency to the implementation of TPT, the 2018 United Nations High-Level Meeting (UNHLM) set ambitious targets, committing to provide TPT to 4 million children <5 years old and 20 million household contacts ≥5 years old [3].

Initial progress toward the UNHLM targets has been derailed by the global COVID-19 pandemic, where efforts to improve TPT implementation were refocused on COVID-19 prevention and treatment [4]. From 2018 to 2020, only 1.2 million household contacts aged <5 years (29% of target) and 320,000 household contacts aged ≥5 years (1.6% of target) were initiated on TPT [1,5]. The initial momentum was lost; TPT uptake among household contacts had been rising from 2015 to 2019, but decreased by 11% in 2020 [6]. Furthermore, delays in TB diagnosis may further increase household transmission [7]. More work is needed to expand TPT implementation among household contacts. This manuscript reviews the evidence for contact investigation and TPT among household contacts, challenges to their implementation, and future directions. 

### 1.1. Risk of TB among Household Contacts 

The burden of TB infection remains high globally and is the highest in household contacts. Prevalence surveys estimate that nearly a quarter of the world’s population is infected [8,9]. In high-burden settings, the prevalence is higher among household contacts, 37.1% among those < 5 years old, 50.2% among those aged 5–14-years, and 42.7% among those aged ≥15 years [10]. Household contacts are also at a high risk of developing TB disease; the pooled prevalence is 3.9% among children <5 years of age, 2.4% among children 5-14 years old, and 5.2% among persons ≥15 years old. The incidence of TB among household contacts is the highest (2%) in the first year following exposure and then declines in subsequent years [10]. 

Household contacts are, therefore, prioritized for the systematic evaluation of TB infection and disease (contact investigation). If contact investigation is not linked to TPT services, household contacts with TB infection may later progress to disease, thereby limiting the effectiveness of contact investigation [11,12].

### 1.2. Impact and Cost-Effectiveness of TB Prevention among Household Contacts 

Analyses of TPT for child contacts have consistently concluded that it is impactful and cost-effective [13,14]. Limited evidence indicated that TPT for adult contacts is likely to be highly impactful. Two modeling analyses showed that contact investigation programs linked to TPT initiation could avert 11–16 persons with TB per contacts aged ≥5 years with and without TB infection and 28–50 persons with TB per contacts aged ≥15 years with TB infection, depending on the TPT regimen and screening algorithm used [15,16]. One study also included secondary transmission impacts and estimated that, in Southeast Asia, TPT for 100% of household contacts of all ages could reduce annual population-level TB incidence by 2–21% and TB mortality by 0.1–9% in >15 years (depending on the country), even if treatment outcomes were to improve [16]. 

Little evidence is available on the cost-effectiveness of TPT for older household contacts, who might be a less cost-effective population to cover than child contacts because of their lower TB mortality and TB incidence [17]. Several studies modeled the cost-effectiveness of TPT for a general population of adults with TB infection, but these cost-effectiveness estimates are of limited applicability to adult household contacts because they do not incorporate the costs of contact investigation, the benefits of finding people with TB during contact investigation, and the implications for both costs and health outcomes of uninfected household contacts, which dilutes the impact and adds costs of either TB infection testing or TPT for those without TB infection [16,18,19,20].

### 1.3. WHO Recommendations for Screening and Treatment of TB Infection in Household Contacts 

**Figure 1:**
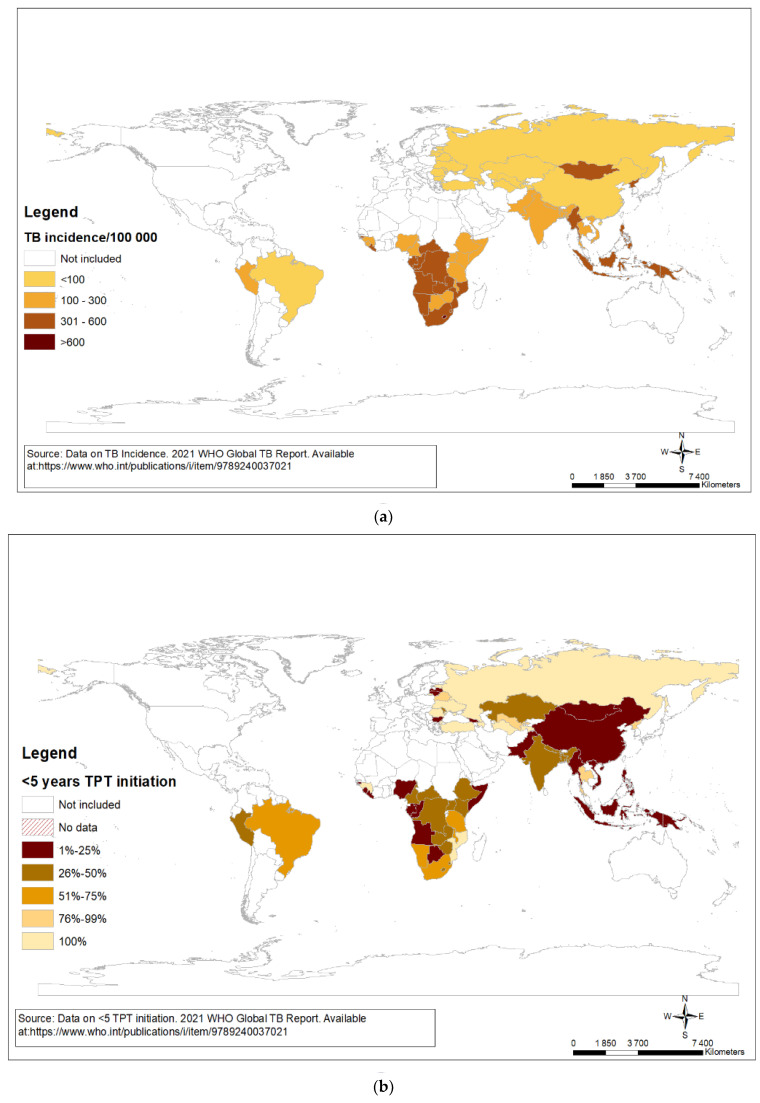
(**a**) TB incidence in high-burden countries for TB, TB/HIV, and drug-resistant TB. (**b**) Percentage of children (aged <5 years) household contacts of bacteriologically confirmed persons with TB on preventive treatment in high-burden countries for TB, TB/HIV, and drug-resistant TB.

Historically, TPT was offered to household contacts <5 years of age given their high risk of progression to TB disease, including severe and disseminated disease. In high-burden settings where TB incidence remains high, TPT uptake remains low (Figure 1a,b). In 2018, the WHO made a conditional recommendation to provide TPT to all household contacts of all ages given their substantially higher risk of progression to TB disease than general population [2]. While testing for TB infection is desirable, treatment may be justifiable without testing based on the risk of exposure and risk of progressing to TB disease in a given setting.

### 1.4. Country TPT Guidelines and Policies 

Following the release of these guidelines, many countries extended the provision of TPT from children <5 years to children <15 years of age [5]. Fewer countries extended this recommendation to adults or other close contacts. Despite these recommendations, TPT implementation remains poor among contacts of all ages [1]. 

### 1.5. TPT Regimens

For several decades, self-administered isoniazid for 6–12 months was the mainstay of preventing TB in persons at high risk, including household contacts. Isoniazid is known to reduce the risk of progression to TB disease by 60% in the 2 years after exposure [21]. Concerns around the hepatotoxicity and poor adherence to long durations of therapy limited its acceptability amongst policy makers, providers, and patients. 

Several rifamycin-based therapies used as single agents or in combination with isoniazid are now recommended by the WHO. Rifampicin can be given alone for 4 months (4R) or with isoniazid for 3 months (3HR). The availability of dispersible, fixed-dose combination HR tablets makes this an attractive option for young children. While 4R is available, it is not widely used due to concern for rifampicin resistance in subclinical disease [22]. Rifapentine can be given in combination with isoniazid either weekly for 3 months (3HP) or daily for one month (1HP). Large, multi-country clinical trials established the efficacy of these short regimens and demonstrated that these regimens had better safety profiles and treatment completion rates than 9-month isoniazid [23,24,25,26,27]. The studied populations and the relative advantages and disadvantages of each regimen are shown in Table 1. While great advances have been made in shortening TPT regimens, evidence is not yet available to inform dosing or safety in some of the highest-risk populations, including children and pregnant women with and without HIV. Furthermore, while rifamycins are the preferred TPT option, known drug–drug interactions with dolutegravir, recommended as first-line antiretroviral therapy, was only studied for 3HP in adults with HIV [28]. 

### 1.6. TPT Durability 

Few data exist on the need to repeat TPT regimens with repeated exposures. In high-burden settings, repeating TPT annually did not provide additional benefits to PLHIVs on antiretroviral therapy [27]. Few data exist to inform the management of household contacts with repeat TB exposures. Many experts repeat a course of TPT for contacts at a high risk of progression to TB disease if a second household member later develops infectious TB. 

### 1.7. TPT Completion and Adherence among Household Contacts 

A meta-analysis of 70 cohorts and nearly 750,000 children and adults with TB infection, predominantly from high-income countries, demonstrated that only 18% of those who initiated completed isoniazid preventive therapy (IPT) versus 49% for a rifamycin-based short-course regimen [29]. A systematic review of child contact management in high-TB-burden countries showed that TPT completion varied from 0 to 95%, with more than half of studies reporting <50% of completion for IPT [30]. Factors associated with TPT completion included: low medication costs, low transportation costs, shorter regimen, directly observed therapy by either a healthcare worker or a community supporter, and initiating therapy at a rural site [30]. Little is known about TPT treatment completion among adolescent and adult household contacts in low- and middle-income countries (LMICs) [1]. While short-course regimens offer an opportunity for improved TPT completion, much work needs to be performed to improve other health-system-, provider-, and patient-level barriers to achieve high levels of TPT completion. 

## 2. Policy-to-Implementation Gap 

### 2.1. Conceptual Mapping of Challenges to TPT Delivery among Household Contacts 

**Figure 2:**
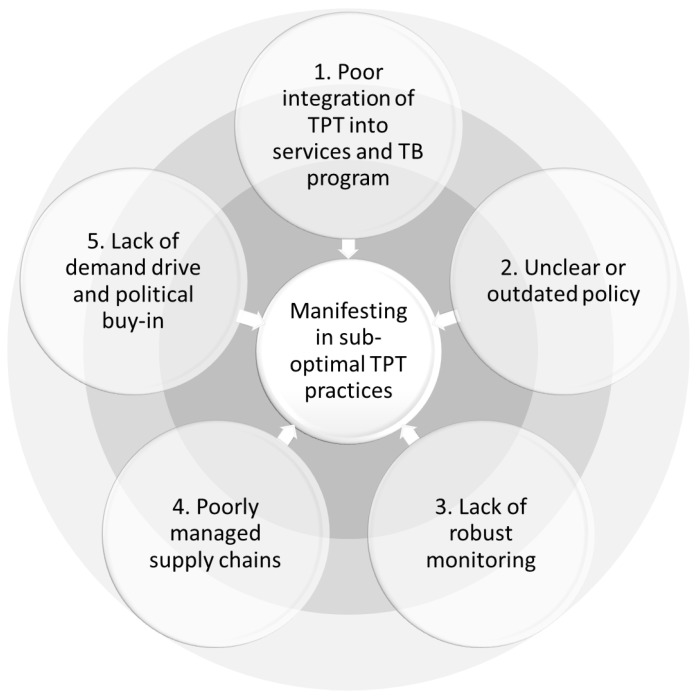
Conceptual mapping of challenges to TPT delivery among household contacts.

The challenges to TPT implementation are complex, with multiple layers of intersecting and synergistic barriers. We propose five linked drivers that underly these challenges (Figure 2). These drivers are relevant throughout the TB prevention cascade of care and manifest in sub-optimal practices that converge at every health service level, from TPT policymaking to patient interaction with the healthcare system.

### 2.2. Driver 1—Poor Integration of TPT into TB Service Delivery Specifically and Primary Care Generally

TPT services and policy are unnecessarily complex and poorly integrated into relevant programing, including both TB and non-TB care. There is lack of clarity about responsibilities for implementing contact tracing and investigation between cadres of facility- and community-based health workers. These TPT service challenges are further compounded by underlying weaknesses of general healthcare services. In the context of multiple disease burdens and limited health service resources, a prevalent attitude that TPT is of lower priority than delivering treatment to patients who are ‘actually sick’ is a barrier to implementation at the provider level. 

### 2.3. Driver 2—Unclear or Outdated Policy and Lack of Appropriate Provider Training

TPT policy at a country, district, or locality level should be regularly updated and cover all aspects of the care cascade (particularly eligibility and screening) and should include specifics in terms of regimen, dosing, and duration for appropriate risk groups, including children and PLHIVs. Policy should also specify the level and area of the health system that takes ownership of contact investigation and TPT implementation. Training for providers should, therefore, flow clearly from policy. Key concepts that are often missing or inadequately addressed in training include messaging around exposure risk (dependent on index patient characteristics), the risk of progression to active TB disease (dependent on individual characteristics and comorbidities), the protective effect of TPT, factors affecting eligibility (e.g., alcohol use), the risk of resistance, the risk of side effects, and resources needed to rule out TB disease. For example, even though tests of infection are no longer part of TPT algorithms, the lack of availability of such tests is still presented as a barrier to TPT implementation, indicating wider policy and practice confusion. Additional implementation considerations that could be addressed both through policy and training include concepts of family-centered adherence (e.g., in household with multiple contacts), addressing stigma and other complex treatment issues (e.g., the concept of ‘pill burden’), and updated definitions of a TB ‘contact’ (which may include close associations outside of the household). 

### 2.4. Driver 3—A Lack of Robust TPT Monitoring Systems with an Over-Focus on Total TPT Initiations rather than Treatment Completions

There is often a lack of or only the most basic indicators for the TPT program. At the facility level, gains in transitioning TB services from siloed, paper-and-pencil TB registers toward integrated data monitoring systems are the least likely to include the TPT program, and the lack of TPT integration into primary care (Driver 1) results in indicators collected in multiple clinical areas (e.g., HIV, TB, pediatrics), which may not be correctly combined to represent the total TPT uptake in a facility. The results are the inadequate allocation of resources to TPT implementation, neglected TPT adherence support, poorly planned and coordinated TPT service needs (e.g., child vs. adult), and ultimately the provider prioritization of other tasks that are adequately monitored. Appropriate TPT monitoring and evaluation systems can help to identify areas of inadequate implementation with focused solutions, including provider training or the resolution of supply-chain bottlenecks. 

### 2.5. Driver 4—Poorly Managed Supply Chains

Regimen, formulation, and service implementation options can be tailored to different patient preferences and needs but may not be available due to medication availability at local, district, and regional levels. Intersecting with Drivers 1 and 2, this also leads to increasingly complex and changeable policy and practice that further delays TPT implementation and oversight. For example, while a short-course regimen may be dramatically more acceptable and would better support adherence, the option is determined by formulation availability and systemic-level influences and not patient-level needs.

### 2.6. Driver 5—A Lack of Demand Drive, Political Buy-In, and Civil Society/Activism for TPT Services

Buy-in from multiple stakeholders can most readily be measured by the availability of funds for implementation. In 2020, of a total of 5.3 billion USD available globally for essential TB services, <0.1 billion USD was earmarked for TB prevention and only covered drugs. By risk group, available spending was skewed heavily towards PLHIVs. This level of funding, driven by multiple stakeholders, is simply inadequate to meet the needs of those at risk globally. The total annual funding required for the successful implementation of TB prevention estimated by the UN High-Level Meeting is 30 million USD, of which only 1/3 was achieved in 2020. For the maximum global public health benefits to be realized from this highly effective intervention, funding for and the prioritization of TPT at the national level must be increased dramatically. 

## 3. Future Directions: How Do We Close the Policy-to-Implementation Gap?

Closing the policy-to-implementation gap requires addressing these five drivers. Below, we list the practical steps to addressing these drivers and achieving improved programmatic outcomes. 

### 3.1. TPT Regimens and Availability 

The high cost of rifapentine has been a deterrent to scaling up HP-based short-course TPT regimens. In 2019, the price of 3HP was successfully reduced to 15 USD per course, thereby allowing the large-scale implementation of 3HP in high-burden countries [31]. To ease patient and health-system burdens, a fixed-dose combination of rifapentine and isoniazid was developed with similar pricing for 3HP (USD 14.25) and 1HP (<USD 20) [32]. Children-friendly formulations of rifapentine are in development but are not anticipated before 2024, thereby limiting access to these short-course regimens for young children. In 2020, N-nitrosamine impurities were identified in rifapentine, halting manufacturing, thereby slowing the implementation of 3HP. Manufacturers are working to remediate their manufacturing processes. 

### 3.2. TB Infection Testing

The programmatic use of testing for TB infection is limited in LMICs. Though not mandated by the WHO, testing for TB infection may be useful to identify persons who would benefit the most from TPT [33] and reassure healthcare workers. A new class of antigen-based skin tests improved specificity over TST but may not otherwise alleviate barriers to TST testing in LMICs [34]. Tests that can accurately predict the risk of progression to disease may also allow targeted preventive treatment to be performed. The CORTIS trial evaluating a blood-based RNA biomarker for TB discriminated between people at a high risk of TB disease but did not predict who would have benefited from TPT [35]. While additional biomarker work is needed to distinguish clinical from subclinical disease and to identify longer-term reactivation risk, studies such as these hold promise to improve access to biomarker screening at the community level [35,36]. 

### 3.3. Approaches to Contact Screening and Investigation

Symptom screening coupled with TB testing using either smear microscopy or Xpert MTB/RIF (Xpert) has been the mainstay for TB diagnosis among household contacts; however, symptom screening is a subjective measure and does not identify subclinical disease. A review of 12 national prevalence surveys in Asia found that 40–79% of bacteriologically confirmed patients did not report TB symptoms [37]. Chest X-ray testing has value in detecting subclinical TB disease, but limited availability remains a significant barrier to its widespread implementation for contact investigation in many high-burden settings [38]. 

Universal Xpert testing of all persons at risk in the clinic setting, including TB contacts, showed a high yield of TB, regardless of symptoms [39,40,41,42]. In South African contacts, there was high acceptability among clinic patients, with 80% uptake, and results revealed a high TB prevalence among asymptomatic persons [40]. Universal TB testing is now implemented among household contacts in South Africa and is under evaluation in Lesotho and Tanzania [43,44]. 

### 3.4. Models of Care for TPT Provision to Household Contacts

Traditionally, TPT is provided in a facility-based model where TB index patients are asked to bring their household members to the clinic for contact investigation and TPT. Transportation cost, lost income, stigma, feeling relatively well, and fear of both long wait times and exposure to TB or other infectious diseases, often persuade household members not to attend clinic. Simple reminders to bring contacts to clinics, including phone calls and letters, may be one effective approach to enhancing contact identification and screening [45,46,47]. Community-based approaches offer an alternative to improving household contacts’ engagement in TB preventive care. Contact investigation, including TB symptom screening and sputum collection, is already accomplished through integrated community-based approaches followed by clinic referral for TPT services. These hybrid approaches have been effective, as 50% more contacts are identified with a community-based versus facility-based approach [48], but are often not linked to TPT services. Only a modest increase in cost would be expected when integrating contact investigation into existing community-based TB services. Cohort studies of community-based TPT initiation in Gambia and Eswatini both demonstrated high TPT acceptability and completion [49,50]. The effectiveness and cost effectiveness of purely community-based models of care, with identification, screening, initiation, and follow-up occurring at the household level, are currently under evaluation using both nurses and community health workers to initiate and follow children on TPT [51,52].

### 3.5. Expanding TPT Services

Promoting TPT for all household contacts instead of just those less than five years of age may increase acceptance among caregivers and facilitate their support to their children to complete TPT. It also an essential step to make these TPT services available to adolescents, a group that is often neglected in healthcare systems. 

Contact tracing has largely focused on household contacts, but other close contacts who do not live in the household may also be at significant risk. Limited evidence suggested that there is a higher proportion of linked transmission outside than inside the household when using genotypic analyses [53]. A pediatric study from Gambia found that only half of children with TB disease were identified when contact tracing was restricted to symptom screening and immediate household contacts only [54]. In contrast, a Vietnamese study of contacts of persons with MDR-TB did not report undiagnosed TB among extended contacts [55]. Exploring the reach and yield of contact tracing in the community is needed to maximize program efficiency, resources, and the impact of contact investigation. Concerns about the stigma and disclosure of TB status to community contacts is of great importance and would need to be explored. Tools such as the Prevent TB app are available to map contacts and who should be prioritized for screening. Innovative strategies for countries to expand contact investigation and TPT beyond household contacts should be further investigated.

## 4. Monitoring and Evaluation

In 2020, the WHO defined standardized indicators for measuring each step in the TPT cascade of care (Table 2) [33]. Implementing and reporting on these indicators is essential to monitoring progress and identify gaps in the care cascade. The collection of data for these indicators could be improved by having electronic person-based registers for TPT that can be used at sub-national and national levels. The programmatic implementation and scale-up of TPT would then require the strengthening of each element in the cascade of care at regular intervals to monitor progress. 

## 5. Conclusions

Household contacts are a high-risk group that the WHO prioritizes for contact investigation and TPT. While many questions persist about the optimal implementation of contact investigation and TPT provision, these questions should not be paralyzing. There is sufficient evidence for TPT implementation among household contacts, and TPT has the potential to significantly reduce TB-associated morbidity and mortality. Even still, innovative approaches to contact screening and investigation, coupled with alternative models of TPT delivery to all household and other close contacts, are needed to bend the epidemic and realize the full potential of these tools in reducing the tuberculosis burden.

## Figures and Tables

**Table 1:** Advantages and disadvantages to WHO-recommended TPT regimens.

WHO-Recommended TPT Regimen	Target Population	Regimen Duration and Frequency	Child-Friendly Formulation	Regimen Advantages	Regimen Disadvantages
1HP	Adolescents of 13 years and older and adults with and without HIV	28 days	Available in the next 2–5 years	- High completion rates; - Lower risk of hepatoxicity and other adverse events compared with 9H.	- No dispersible RPT tablet for children available; - No dosing for children <13 years old; - Market size does not support fixed-dose combination; - Not studied in pregnant women.
3HP	Children of 2 years and older, adolescents, and adults	12 weekly doses	Available in the next 2–5 years	- High completion rates; - Lower risk of hepatoxicity and other adverse events compared with 9H.	- No dispersible RPT tablet for children available; - No dosing for children <2 years old; - Market size does not support fixed-dose combination; - High pill burden per dose, lower overall pill burden; - Challenging to remember weekly dose; - Safety during pregnancy not known.
3RH	Persons of all ages with and without HIV	90 daily doses	Currently available as dispersible FDC for children of <25 kg	- Preferred regimen for young children without drug–drug interactions; - Co-formulated with isoniazid into a dispersible tablet; - High completion rates; - Low risk of hepatoxicity and other adverse events.	- Drug–drug interactions with oral contraceptives, ARVs including DTG, LPV/r, and NVP (prophylaxis and treatment); - Not studied in pregnant women for prevention.
4R	Persons of all ages with and without HIV	120 daily doses	Can be compounded into syrup, not readily available in LMICs	- High completion rates; - Low risk of hepatoxicity and other adverse events.	- Limited availability of rifampicin without coformulation; - Can be compounded into suspension, but requires specialized skills; - Limited evidence to support dose adjustment in young children living with HIV on DTG; - Drug–drug interactions with oral contraceptives, ARVs including DTG, LPV/r. and NVP (prophylaxis and treatment); - Not studied in pregnant women for prevention.
6–9H	Persons of all ages with and without HIV	180–270 daily doses	Dispersible tablet available	- Low cost; - Few drug–drug interactions; - Only regimen that can be used in CLHIVs on DTG; - Dispersible tablet available.	- 6–9 month duration associated with poor completion rates; - Adverse events perceived to be too high; - Increased adverse pregnancy outcomes when used in the second or third trimester.

1HP = daily rifapentine and isoniazid for 1 month; 3HP = weekly rifapentine and isoniazid for 3 months; 9H = daily isoniazid for 9 months; 3RH = daily rifampicin and isoniazid for three months; 4R = daily rifampicin for 4 months; RPT = rifapentine; LMICs = low- and middle-income countries; ARV = antiretroviral therapy; DTG = dolutegravir, LPV/r =Lopinavir/Ritonavir; NVP = Nevirapine; CLHIVs = children living with HIV.

**Table 2:** WHO-recommended indicators for measuring TPT cascade of care.

Indicator	Definition	Calculation	Notes
Contact investigation coverage	Percentage of contacts of bacteriologically confirmed pulmonary TB who are evaluated for TB disease	Numerator: number of contacts of bacteriologically confirmed pulmonary TB disease who completed evaluation for TB disease (+/− TB infection) Denominator: number of contacts of bacteriologically confirmed pulmonary TB disease (+/− TB infection)	Denominator may be estimated using TB notifications and demographic data
TPT coverage	Percentage of TPT-eligible contacts who initiate TPT	Numerator: number of individuals initiated on TPT Denominator: number of TPT-eligible individuals	This can be calculated separately using TPT indication (household contact, PLHIV, etc.) and by age (<5 years old, 5–14 years old, 15+ years old, etc.)
TPT completion	Percentage of individuals who initiated and completed TPT	Numerator: number of individuals completing TPT Denominator: number of individuals initiating TPT	This can be calculated separately using TPT indication (household contact, PLHIV, etc.) and by age (<5 years old, 5–14 years old, 15+ years old, etc.)

TB = tuberculosis; TPT = TB preventive treatment; PLHIVs = people living with HIV.

## Data Availability

Not applicable.
